# Testing the Geometric Series Hypothesis for the Ascending Leaf Length Sequence per Culm in Six *Indocalamus* Bamboo Species

**DOI:** 10.3390/plants15203129

**Published:** 2026-10-13

**Authors:** Yimin Zhu, Weihao Yao, Peijian Shi

**Affiliations:** 1 School of Forestry Science and Technology, Lishui Vocational and Technical College, 357 Xinyuan Street, Lishui 323000, China; yizhum@126.com; 2 College of Landscape Architecture, Zhejiang A&F University, 666 Wusu Street, Hangzhou 311300, China; 3 Bamboo Research Institute, Nanjing Forestry University, 159 Longpan Road, Nanjing 210037, China; whyao@njfu.edu.cn

**Keywords:** Bambusoideae, biogeom package, leaf size, nonlinear regression, Poaceae

## Abstract

The distribution of leaf sizes along a shoot is a fundamental aspect of plant architecture, but a simple quantitative rule for this pattern has not been widely tested. We examined whether the ascending leaf length sequence per culm in six *Indocalamus* bamboo species follows a geometric series. This hypothesis is particularly plausible for *Indocalamus* bamboos because their leaves are concentrated near the culm apex, resulting in relatively uniform light exposure across the leaf cohort. However, the culm architecture is not entirely uniform: some leaves arise from smaller, thinner branches, which could naturally generate a geometric size sequence. To test this hypothesis, we measured the lengths of all leaves from 602 culms across the six species. After excluding culms with fewer than three or more than 90 leaves and those with estimated common ratios exceeding 1.2, we retained 577 culms for final analysis, representing 95.8% of the initial sample. For each culm, we fitted a geometric series model to the ascending leaf length sequence using nonlinear least-squares regression. The goodness of fit was evaluated using the mean absolute percentage error (MAPE) between observed and predicted cumulative leaf lengths. The results showed that the geometric series model fitted the data remarkably well, with 75.39% of the 577 culms having a MAPE below 5% and 96.53% below 10%. The estimated common ratios ranged from 1.01 to 1.19, with a mean of 1.05, indicating a modest but consistent geometric increase in leaf length. Significant differences in both the estimated common ratio and the estimated minimum leaf length were found among species, reflecting interspecific variation in growth strategies. These findings provide strong support for the geometric series as a robust descriptor of leaf length organization per culm in bamboos, offering new insights into the architectural structures that constrain the leaf size distribution per culm.

## 1. Introduction

The size of individual leaves is a fundamental functional trait that profoundly influences light interception, photosynthetic carbon gain, transpirational cooling, and whole plant resource allocation [1,2]. Grasses, including bamboos, represent an ideal system for examining leaf size variation because of their global ecological dominance and distinctive leaf morphology [3,4]. A global analysis of leaf size across species and climatic gradients has shown that smaller leaves are consistently associated with colder and drier environments, reflecting adaptive tradeoffs between boundary layer conductance, hydraulic safety, and developmental constraints [4]. At the within-plant level, previous studies have focused predominantly on the scaling relationships between leaf area and leaf mass, demonstrating a pervasive “diminishing returns” pattern, in which larger leaves incur disproportionately greater biomass investment per unit area [5,6]. Similarly, the allometric relationships between leaf area, length, and width have been examined in diverse taxa, including bamboos, to understand how leaf shape influences the proportional relationship between leaf area and the product of length and width [3,7,8,9]. The tradeoff between leaf size and the number of leaves per shoot has also been investigated, with maximum individual leaf size scaling negatively with leafing intensity [10,11]. Despite these extensive efforts, however, the quantitative rules constraining the leaf size sequence within a single shoot, when leaves are ordered by size rather than by spatial position, have received remarkably little attention. Most existing studies treat leaf sizes as independent observations drawn from a common distribution and analyze their aggregate statistical properties, such as the mean, variance, or coefficient of variation, without considering whether the rank-ordered sequence itself follows a predictable mathematical pattern. This oversight is notable because recent evidence has shown that leaf size sequences, when ordered by size rather than by spatial position, can follow a simple mathematical rule [12], raising the possibility that such ordered sequences may reflect underlying developmental or architectural constraints on organ size variation [13].

Traditional approaches to quantifying leaf size variation within a shoot have relied predominantly on dispersion metrics, such as the coefficient of variation and the generalized entropy index, which measure relative spread but provide no information about the shape of the size sequence or the rank order in which leaves appear [14,15]. More recently, the Gini coefficient, derived from Lorenz curve analysis, has been applied to leaf area distributions to quantify the degree of inequality in resource allocation among leaves within a culm or plant [16,17,18,19]. These distribution-based methods, including recent work on fitting Gamma and Weibull functions to bamboo leaf area distributions [19], offer valuable insights into the overall heterogeneity of leaf sizes. Nevertheless, these approaches treat leaf sizes as an unordered set and focus on the aggregate properties of the distribution, rather than on the ordered relationship among individual leaf sizes. Recent studies have shown that geometric series can describe sorted size sequences of floral parts and leaves [20], and have further confirmed this pattern for tepal-length sequences in a *Magnolia* species [21]. However, for leaf size sequences, the fit of the geometric series model was considerably less accurate than for floral parts, with mean absolute percentage errors approaching or exceeding 10% in species with vertically distributed leaves [20]. This observation raises the question of whether leaf arrangement architecture influences the applicability of the geometric series, and whether species with different leaf deployment patterns might yield closer adherence to this model.

**Figure 1:**
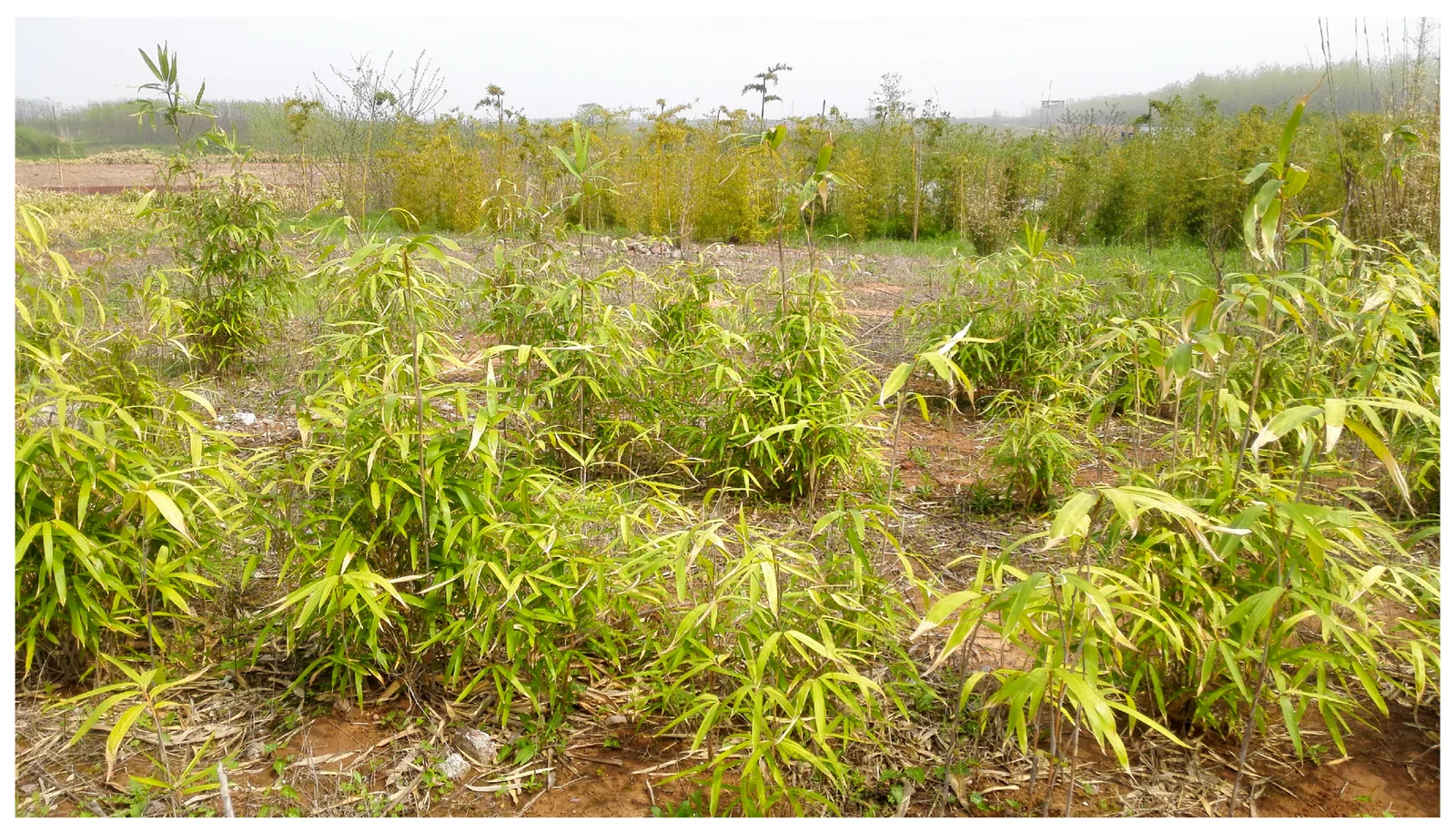
Representative clustered shoots of an *Indocalamus* species (*I. barbatus*).

The bamboos of the genus *Indocalamus* provide an unusually suitable system for testing the geometric series hypothesis for leaf size sequences. Unlike many woody plants with leaves distributed along complex branching systems, *Indocalamus* leaves are predominantly clustered near the apex of each culm (Figure 1), resulting in relatively uniform light exposure across the leaf population [22]. This apical concentration minimizes the vertical light gradient that complicates interpretations in species with leaves vertically distributed along the culm, such as *Semiarundinaria densiflora*, where shade-induced morphological plasticity can obscure intrinsic size-ordering patterns [19,23]. In contrast, the apical clustering in *Indocalamus* creates a more homogeneous light environment, making it easier to detect whether leaf size sequences follow a simple multiplicative rule. However, the architectural structure is not entirely uniform: some leaves are borne on smaller, thinner branches, which could naturally generate a proportional size sequence among leaves. To test this prediction, we measured the lengths of all leaves from 602 culms across six *Indocalamus* species. For each culm, we fitted a geometric series model to the ascending leaf length sequence using nonlinear least-squares regression protocols and evaluated the goodness of fit using the mean absolute percentage error between observed and predicted cumulative leaf lengths per culm. This study tests whether the geometric series, which has been validated for floral part size sequences, can also describe the leaf length distribution per culm in *Indocalamus* bamboos, and whether the common ratio of the sequence serves as a useful measure of leaf size organization. The present study contributes to a broader understanding of how plants organize repeated organs along a size continuum, independent of spatial position, and provides a quantitative approach for comparing size ordering patterns across different plant groups.

## 2. Materials and Methods

### 2.1. Leaf Sampling and Data Acquisition

To cover a representative range of the genus *Indocalamus* Nakai, we selected six species across three locations. Four species (*I. barbatus*, *I. pedalis*, *I. pumilus*, and *I. victorialis*) were collected from the Xinzhuang Campus of Nanjing Forestry University (32°5′00″ N, 118°48′47″ E, 23.7 m a.s.l.) in early July 2014, with 90 culms for each species. The remaining two species were collected in Sichuan Province in November 2024: 122 culms of *I. decorus* from Hongya County (29°55′33″ N, 103°27′53″ E, 504.5 m a.s.l.) and 120 culms of *I. longiauritus* from the Leshan Normal University Campus (29°33′54″ N, 103°44′57″ E, 419.6 m a.s.l.). The same sampling criteria and measurement procedure were applied in both sampling periods. Culms were selected based on developmental maturity, and all sampled culms were at a comparable mature vegetative stage. Healthy culms were defined as those showing normal growth without obvious disease symptoms or severe pest damage. Leaves were considered mature when the blades were fully expanded and normally green. Leaves showing clear senescence, such as marked yellowing or browning, or damage that substantially affected lamina integrity were excluded. For each species, culms meeting these criteria were selected randomly at the corresponding sampling site. Individual culms were used as the sampling and analytical units throughout the study. For each leaf, lamina length was measured as the distance from the junction between the pseudo-petiole and the lamina to the leaf apex and was recorded with 0.1 cm precision. The same measurement protocol was applied to all six species. All leaf length data are accessible online from Supplementary Table S1.

### 2.2. Data Analysis

To test the validity of the geometric series (GS) hypothesis for the leaf length distribution per culm, we sorted the leaf lengths in ascending order for each culm and used the formula for the sum of the first *j* terms (1 ≤ *j* ≤ *n*, where *n* is the total number of leaves per culm) of a GS (denoted as *S_j_*) to compare the observed cumulative leaf length from the smallest leaf to the *j*th leaf in the ascending leaf length sequence for each culm. In a geometric series, the cumulative length of the first *j* terms (i.e., $\sum_{u=1}^{j}L_{u}$) takes the form:

(1) $$S_{j}=\sum_{u=1}^{j}L_{u}=L_{1}\frac{1-q^{j}}{1-q},$$

where *q* is the common ratio of the GS and *L*_1_ is the first term of the GS, i.e., the length of the smallest leaf of a culm. If the leaf length sequence follows a GS, the numerical value of the common ratio (*q*) quantifies the degree of variation in leaf lengths.

If a sorted leaf length sequence in ascending order follows a GS, each term can be expressed as $L_{i}=L_{1}q^{i-1}$ (*i* = 1, 2, 3, …, *n*), where *L_i_* and *i* represent dependent and independent variables, respectively, and *q* and *L*_1_ are the model parameters to be estimated [24]. For each culm, the observed data consist of $\left\{i,L_{i}\right\}$ (*i* = 1, 2, 3, …, *n*). The geometric series model was fitted using nonlinear least-squares regression protocols by minimizing the residual sum of squares with the Nelder–Mead algorithm [25]. The initial value of *q* was set to the mean of the quotients of adjacent terms in the sorted leaf length sequence. For each term *L_i_*, a provisional first term was calculated as $L_{i}/q^{i-1}$, and the initial value of *L*_1_ was taken as the mean of these *n* provisional values [24]. Nonlinear least-squares regression was performed using the “fitGS” function of the “biogeom” package (version 1.5.2) [26] in R (version 4.3.3) [27] (see Appendix A for an example of its implementation). The estimates of the common ratio and the first term of the hypothesized GS were represented as $\hat{q}$ and ${\hat{L}}_{1}$, respectively. Substituting the estimates of the two model parameters into Equation (1), we calculated the theoretical (predicted) cumulative leaf length for the first *j* leaves of the ascending leaf length sequence (denoted as ${\hat{S}}_{j}$).

The mean absolute percentage error (MAPE) between the observed and predicted cumulative leaf lengths was computed for each culm to evaluate the goodness of fit of Equation (1):

(2) $$MAPE=\frac{1}{n}\sum_{j=1}^{n}\left|\frac{\sum_{u=1}^{j}L_{u}-{\hat{S}}_{j}}{\sum_{u=1}^{j}L_{u}}\right|\times 100\%,$$

where ${\hat{S}}_{j}$ represents the predicted cumulative leaf length for the first *j* leaves (i.e., the sum of leaf lengths from the smallest leaf to the *j*th leaf, where *j* = 1, 2, 3, …, *n*) in a culm based on Equation (1). As a rule of thumb, a MAPE value below 5% indicates a good fit [20,24]. To avoid potential smoothing of individual-leaf deviations by cumulative leaf lengths, model performance was additionally evaluated using individual leaf lengths. For each culm, the root-mean-square error (RMSE) and MAPE between the observed and predicted individual leaf lengths were calculated.

Before the primary analysis, culms with fewer than three leaves were excluded because the GS model contains two fitted parameters (*L*_1_ and *q*), making goodness-of-fit evaluation with only two observations uninformative. Extreme observations were subsequently identified based on leaf number (*n*) and the estimated common ratio ($\hat{q}$). Initial analysis showed that only one culm had *n* > 90 (*n* = 91; Figure S1A). After excluding culms with fewer than three or more than 90 leaves, 592 culms remained; among these, only 15 (2.5%) had $\hat{q}$ > 1.2, forming a sparse right tail in an otherwise strongly right-skewed distribution of $\hat{q}$ (Figure S1B). Because $\hat{q}$ > 1.2 implies an average increase of more than 20% between successive ranked leaves, such values were regarded as extreme relative to the predominant pattern. Culms with *n* > 90 or $\hat{q}$ > 1.2 were excluded from the primary analysis. To assess the effects of these exclusions on model performance, sensitivity analyses were conducted by reintroducing the culm with *n* > 90, the culms with $\hat{q}$ > 1.2, and both sets of observations.

Pairwise comparisons among the six species for the observed minimum leaf length (*L*_1_), the coefficient of variation in leaf lengths (CV*_L_*), the estimated first term (${\hat{L}}_{1}$, i.e., the estimated minimum leaf length), and $\hat{q}$ were performed using Tukey’s honestly significant difference (HSD) test at α = 0.05 [28]. The coefficient of variation (CV = SD/mean × 100%) was used to assess intraspecific variation for each of the four variables. The association between $\hat{q}$ and *n* was evaluated separately for each species using Spearman’s rank correlation.

All statistical analyses were conducted using R (version 4.3.3) [27].

## 3. Results

A total of 577 culms from six *Indocalamus* species were retained for the primary analysis after excluding culms with fewer than three or more than 90 leaves, and those with estimated common ratios exceeding 1.2. Of the 602 initially sampled culms, nine contained only two leaves and one contained 91 leaves, leaving 592 culms after the leaf-number screening. Of these, 15 culms had estimated common ratios greater than 1.2 and were excluded from the primary dataset. The distributions of leaf number and estimated common ratio before exclusion are shown in Figure S1, with the excluded observations occurring at the extreme ends of their respective distributions. The geometric series (GS) model provided a good fit to the ascending leaf length sequence per culm for the majority of culms. The mean absolute percentage error (MAPE) was 3.58%, with 75.39% of the culms having MAPE values below 5% and 96.53% below 10% (Table 1). Sensitivity analyses yielded highly similar model-fitting results when the culm with leaf number (*n*) > 90, the culms with estimated common ratios ($\hat{q}$) greater than 1.2, or both sets of observations were reintroduced (Table 1). These results indicate that the exclusion of these extreme observations had little effect on overall model-fitting performance.

**Figure 2:**
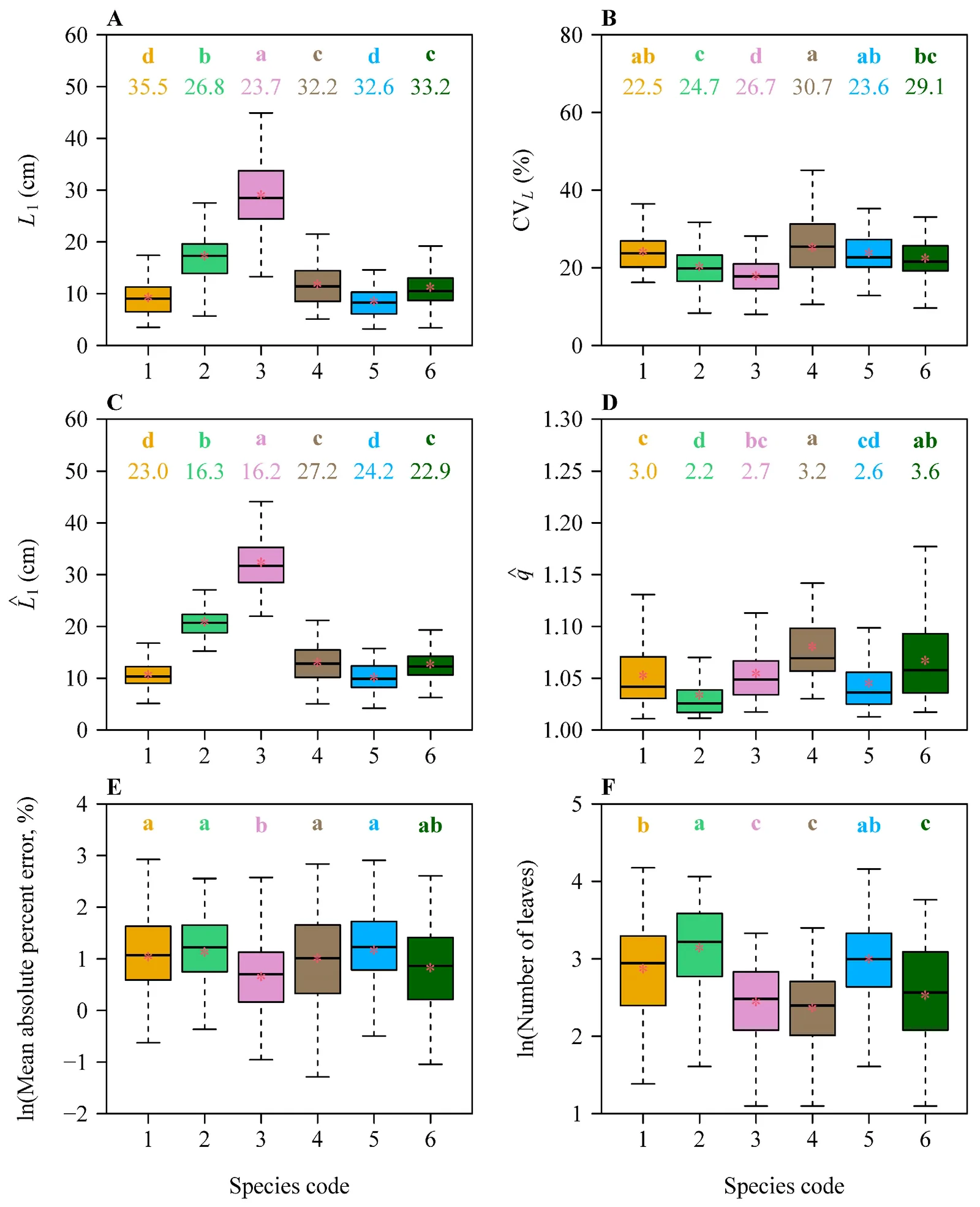
Boxplots of (**A**) the observed minimum leaf length per culm (*L*_1_), (**B**) coefficient of variation in leaf lengths per culm (CV*_L_*), (**C**) predicted minimum leaf length per culm (${\hat{L}}_{1}$), (**D**) estimated common ratio of the leaf length sequence per culm ($\hat{q}$), (**E**) natural logarithm of the mean absolute percent error (MAPE), and (**F**) natural logarithm of the number of leaves per culm for the six *Indocalamus* species. The colored lowercase letters in each panel denote significant differences among species based on Tukey’s HSD test (α = 0.05); colored numbers below the letters are the coefficients of variation (CV in %); thick horizontal lines inside the boxes represent the medians; and asterisks indicate the means. Species codes 1–6 correspond to *I. barbatus*, *I. decorus*, *I. longiauritus*, *I. pedalis*, *I. pumilus*, and *I. victorialis*, respectively.

**Figure 3:**
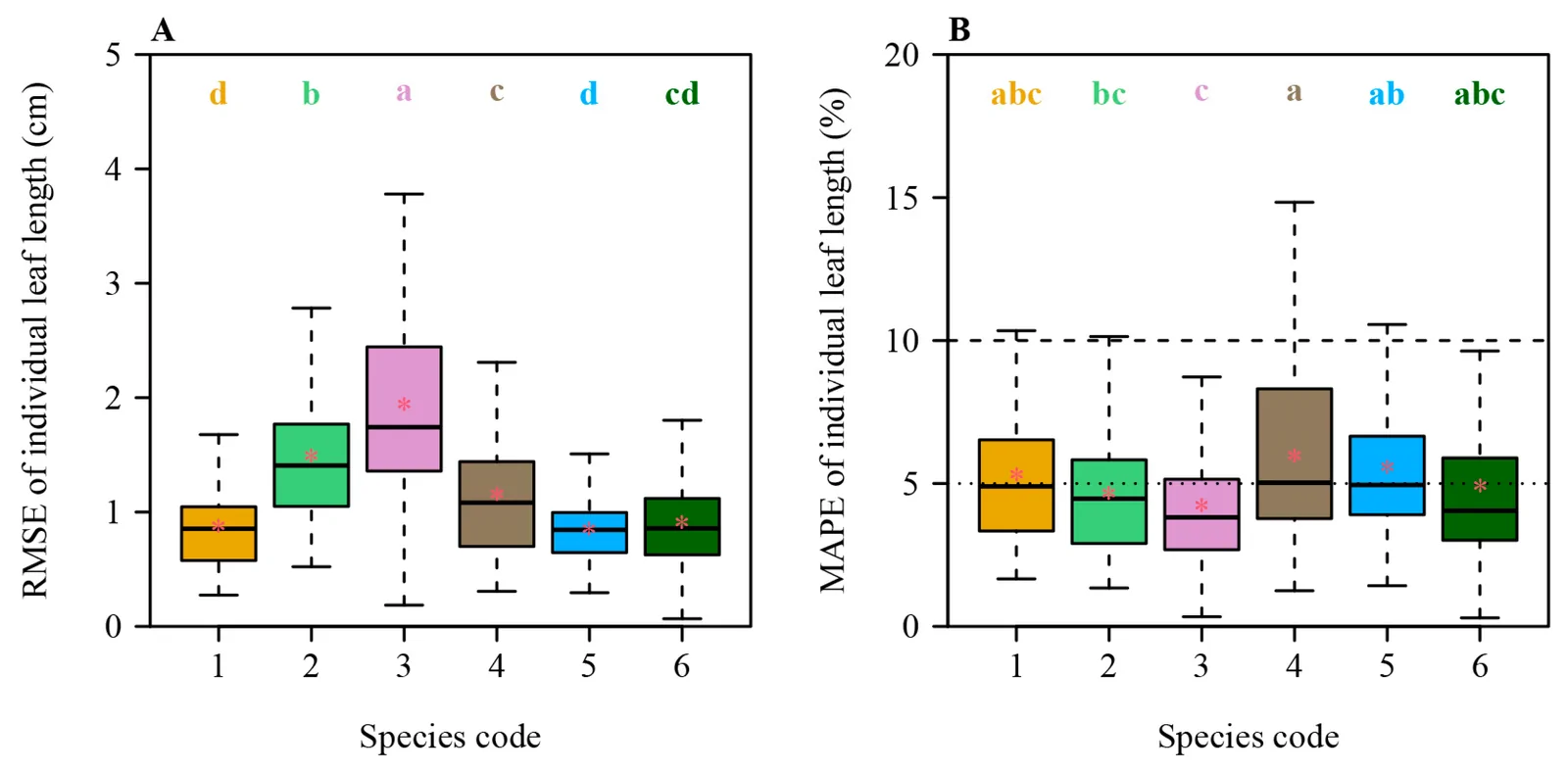
Model-fitting errors evaluated directly from individual leaf lengths of the six *Indocalamus* species. (**A**) Root-mean-square error (RMSE) and (**B**) mean absolute percentage error (MAPE) between observed and predicted individual leaf lengths. The colored lowercase letters in each panel denote significant differences among species based on Tukey’s HSD test (α = 0.05) The horizontal bold lines within the boxes represent the medians, and the asterisks represent the means. In panel (**B**), the dotted and dashed horizontal lines indicate MAPE values of 5% and 10%, respectively.

Pairwise comparisons among the six species indicated significant interspecific differences in all four variables examined (Figure 2). For the observed minimum leaf length (Figure 2A), *I. longiauritus* exhibited the largest values, whereas *I. pumilus* showed the smallest. The coefficient of variation in leaf lengths (Figure 2B) ranged from approximately 3% to 45% across species, with some species showing substantially higher variation than others. The estimated first term (${\hat{L}}_{1}$, i.e., the predicted minimum leaf length; Figure 2C) followed a similar pattern to the observed minimum leaf length, with *I. longiauritus* having the largest estimated minimum leaf length and *I. pumilus* the smallest. The estimated common ratio (Figure 2D) ranged from approximately 1.01 to 1.19 across species, with *I. pedalis* and *I. victorialis* exhibiting the largest mean common ratios and *I. decorus* the smallest. The natural logarithm of MAPE (Figure 2E) was consistently low across all species, with *I. longiauritus* showing the smallest prediction errors. The natural logarithm of the number of leaves per culm (Figure 2F) differed significantly among species. Model performance was further evaluated directly using individual leaf lengths. Across the 577 culms, the mean RMSE and MAPE between observed and predicted individual leaf lengths were 1.25 cm and 5.02%, respectively. In addition, 94.97% of the culms had MAPE values below 10% (Figure 3), indicating that the GS model also showed good fitting performance at the individual-leaf level.

**Figure 4:**
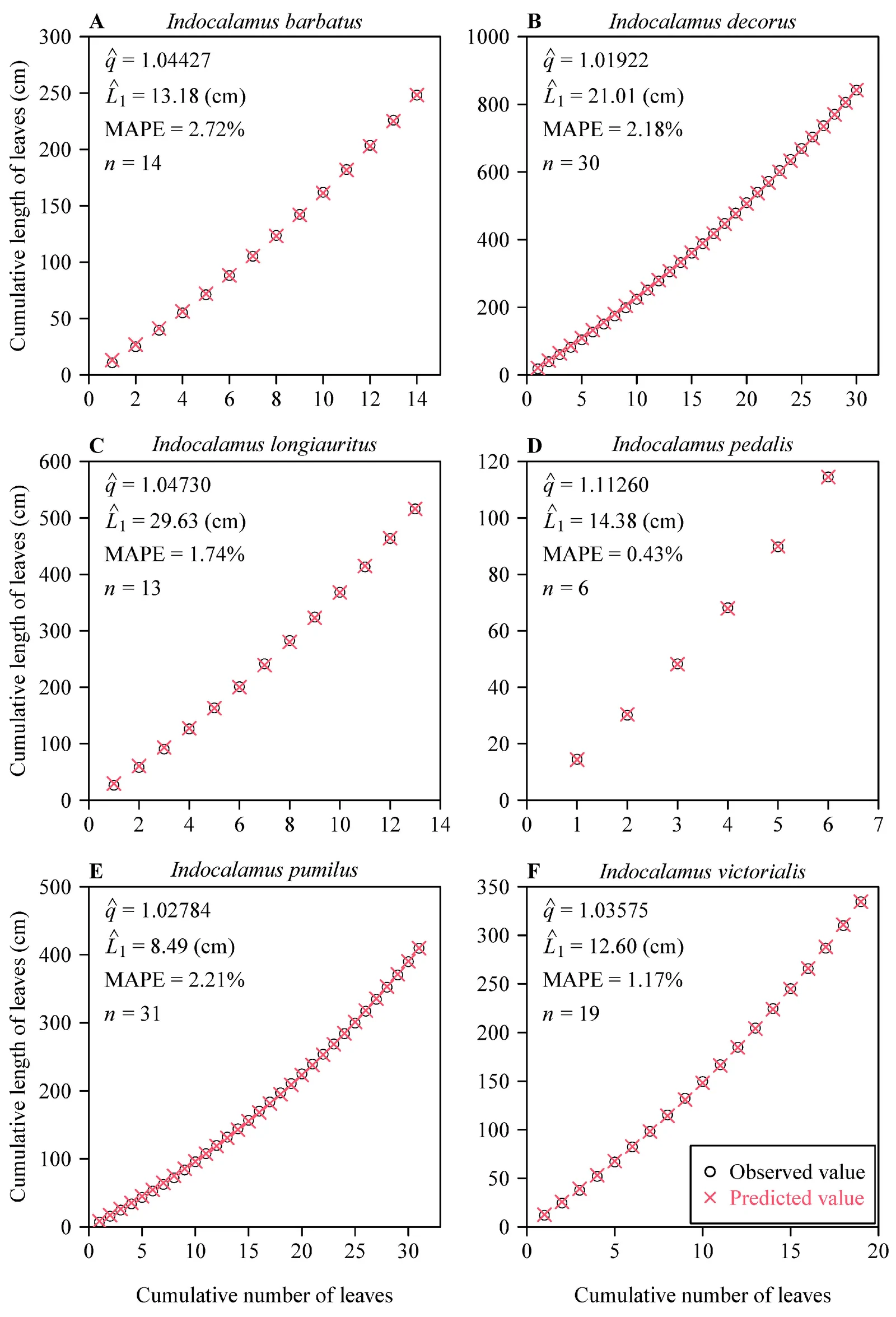
Comparison of the observed and predicted cumulative leaf lengths for the representative culms of the six *Indocalamus* species (**A**–**F**). Open circles represent observed cumulative leaf lengths, and crosses indicate the corresponding predicted values. Here, $\hat{q}$ is the estimated common ratio of the leaf length sequence per culm, ${\hat{L}}_{1}$ is the estimated first term of the leaf length sequence (i.e., the predicted minimum leaf length), MAPE is the mean absolute percent error (in %), and *n* is the number of leaves per culm.

Figure 4 illustrates representative examples of the observed versus predicted cumulative leaf lengths for individual culms from each of the six species. In all cases, the geometric series model closely tracked the observed cumulative leaf lengths from the smallest to the largest leaf, with MAPE values consistently below 3% for the selected culms. The estimated common ratios for these representative culms ranged from approximately 1.01 to 1.12, and the estimated first terms corresponded closely to the observed minimum leaf lengths, confirming the validity of the geometric series model for describing the ascending leaf length sequence per culm.

**Figure 5:**
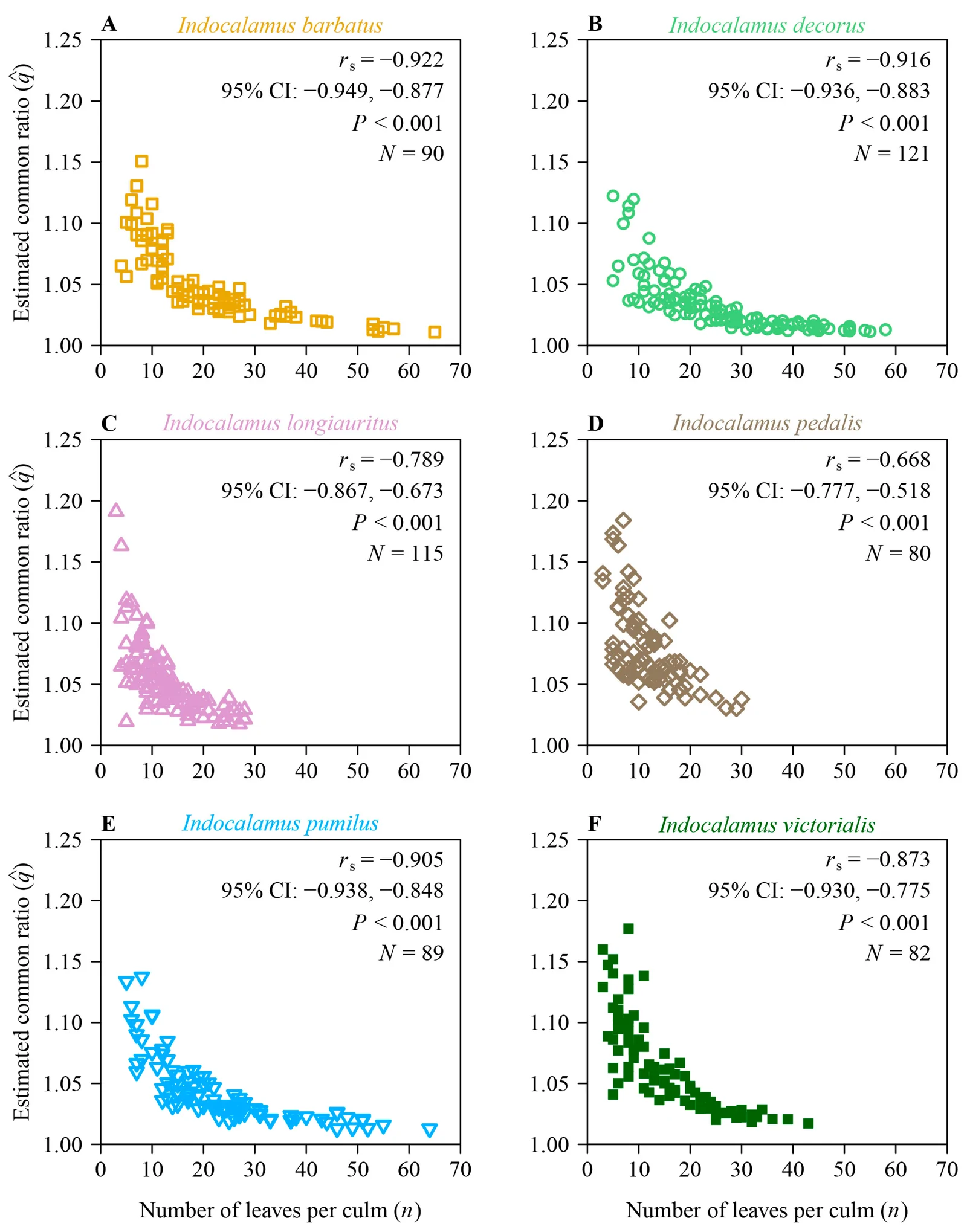
Relationships between the estimated common ratio ($\hat{q}$) and number of leaves per culm (*n*) in the six *Indocalamus* species. Each point represents one culm. Spearman’s rank correlation coefficient (*r*_s_), its 95% confidence interval (CI), *p*-value, and number of culms (*N*) is shown in each panel. Panels (**A**–**F**) correspond to *I. barbatus*, *I. decorus*, *I. longiauritus*, *I. pedalis*, *I. pumilus*, and *I. victorialis*, respectively.

Significant negative associations between $\hat{q}$ and *n* were detected within all six species (Figure 5). Spearman’s rank correlation coefficients (*r*_s_) ranged from −0.668 to −0.922, and the 95% confidence intervals were entirely below zero for all species. All associations were significant (*p* < 0.001).

## 4. Discussion

The results of the present study provide strong empirical support for the geometric series hypothesis for leaf length sequences within culms of six *Indocalamus* species, with MAPE values below 5% for 75.39% of the 577 culms examined. The estimated common ratios ranged narrowly from 1.01 to 1.19, with a mean of 1.05, indicating a modest but consistent geometric increase in leaf length along the ascending size sequence. Significant differences were observed among the six sampled species in both the estimated common ratio and the estimated minimum leaf length, suggesting variation in leaf size organization among species. Furthermore, significant negative relationships between the estimated common ratio and leaf number were consistently detected across the six species (Figure 5), raising questions about the factors underlying this pattern. In the following sections, we first justify the use of leaf length rather than leaf area as the primary metric for testing the geometric series hypothesis (Section 4.1). We then explore why the estimated common ratio tends to diminish as the number of leaves per culm increases (Section 4.2). Finally, we discuss the limitations of the present study and propose directions for future research (Section 4.3).

### 4.1. Why the Leaf Length Sequence Rather than the Leaf Area Sequence?

The present study examined the leaf length sequence rather than the leaf area sequence to test the geometric series hypothesis for *Indocalamus* bamboos. This choice warrants justification, given that leaf area is the more direct measure of photosynthetic capacity and has been used in prior geometric series studies on floral parts and leaves [20]. Several lines of evidence support the use of leaf length as a suitable metric for examining the ascending leaf size sequence on each culm.

The square-law relationship between leaf area and length in bamboos is closely related to leaf shape. Shi et al. [3] examined over 10,000 leaves from 101 bamboo taxa and found that although the Montgomery equation (assuming an isometric relationship between the leaf area and the product of length and width) applied universally across bamboo taxa, the isometric relationship between leaf area and leaf length squared was influenced by variation in leaf shape. At the pooled level across all taxa, the validity of the leaf area vs. length-squared relationship depended critically on the degree of variation in the ratio of leaf width to length, with a strong positive correlation between this coefficient of variation and the prediction error of the leaf area vs. length-squared fit. However, at the individual species level, the relationship between area and length squared remained valid and reliable for most taxa (Figure 1C published in Shi et al. [3]). This pattern is explained by the remarkable conservation of bamboo leaf shape. Shi et al. [22] demonstrated that a simplified Gielis equation with only two parameters, a size parameter and a shape parameter, described the leaf profiles of four *Indocalamus* species (including those examined here) with good fit, and that the shape parameter, which directly reflects the ratio of leaf width to length, ranged narrowly across species. Lin et al. [29] extended this finding to 42 bamboo species and found that the shape parameter ranged narrowly from 0.02 to 0.10, with an average coefficient of determination of 0.995 for fitting the simplified Gielis equation to bamboo leaf profiles. These results indicate that bamboo leaves share a common lanceolate form with limited interspecific variation in shape. Consequently, a geometric series in leaf length should translate into a geometric series in leaf area at the within-species level, making length a reliable proxy for area in this context.

Empirical evidence from other plant groups further supports the use of length over area for geometric series analysis. Deng et al. [21] demonstrated that tepal length sequences in *Magnolia × soulangeana* conform to a geometric series with high precision, yielding mean absolute percentage errors below 1.2% for all 359 flowers examined. More directly, Xu et al. [30] compared petal length and petal area sequences across nine *Malus* cultivars and found that the petal length sequence per flower fit the geometric series substantially better than the petal area sequence per flower: all 540 flowers had MAPE values below 2.5% for petal length sequences (mean 0.32%), whereas petal area sequences had a mean MAPE of 0.75% and a maximum of 4.52%. This difference likely reflects the fact that length is a one-dimensional measure under relatively tight developmental control, whereas area integrates variation in both longitudinal and transverse dimensions. Importantly, Su et al. [31] showed that for most species investigated, the mean percent error in leaf area did not exceed 5% even when the floating ratio of leaf length ranged from −15% to 0 for bamboo species and from 0 to 20% for the tree species examined, indicating that leaf area is relatively conservative despite considerable variation in leaf length. This finding suggests that leaf area, while often used as a measure of leaf size, may be less sensitive to subtle variations in organ dimensions that could be detected by geometric series analysis of leaf length data. Practically, length measurements are also simpler and less labor-intensive to obtain in the field than area measurements, requiring only a ruler rather than scanning and image analysis. Recent advances in non-destructive phenotyping have also shown that linear dimensions and 2D image-derived traits can accurately estimate leaf area [32]. Thus, the geometric series-based approach, when combined with appropriate relationships between linear dimensions and area or biomass, may contribute to rapid, non-destructive estimation of leaf area or biomass for plant phenotyping and nursery or plantation monitoring.

From a developmental perspective, leaf length represents the primary axis of organ growth, determined by the duration and rate of cell division and expansion along the proximodistal axis. This axis is established early in leaf primordium development and is under relatively stringent genetic and hormonal control. The *ROTUNDIFOLIA3* gene, for example, controls leaf length via the biosynthesis of specific steroids, and its overexpression produces longer leaves without changes in leaf width [33]. In contrast, the *ANGUSTIFOLIA* gene regulates leaf width by controlling polar cell elongation and the arrangement of cortical microtubules, and its mutation produces narrow leaves [34]. Moreover, leaf development involves the coordination of growth along both the proximodistal (length) and mediolateral (width) axes [35]. These axes are regulated by partially independent genetic pathways, with cell division and expansion along each axis being subject to distinct controls [36]. The greater developmental canalization of length compared to width may therefore explain why length sequences exhibit closer adherence to the geometric series and smaller prediction errors when fitted to this model. For these reasons, the mathematical relationship between length and area in bamboos, the developmental stability of length as a trait, and empirical evidence from other plant groups all support the use of the leaf length sequence as an appropriate and practical metric for testing the geometric series hypothesis in *Indocalamus* bamboos.

### 4.2. Why Does the Estimated Common Ratio Tend to Decrease with Increasing Number of Leaves per Culm?

One of the notable patterns emerging from our analysis is the consistent negative relationship between the estimated common ratio ($\hat{q}$) and leaf number (*n*) across all six species (Figure 5). Thus, culms with more leaves generally had smaller common ratios, indicating a more finely graded increase in leaf length across the ordered sequence. One possible biological explanation for this pattern may be related to the leaf arrangement architecture of *Indocalamus* bamboos and the relatively uniform light environment associated with their apically clustered leaves.

For *Indocalamus* bamboos, leaves are predominantly clustered near the culm apex, which may create a relatively homogeneous light environment across the leaf cohort [22,37]. Under such apical clustering, self-shading may be reduced because most leaves are positioned at similar heights and receive comparable irradiance. Consequently, the developmental responses of leaves to light are relatively uniform. However, despite this light uniformity, leaf size is not identical across all leaves. Differences in branch architecture, such as branch thickness and position along the culm, may contribute to variation in hydraulic conductance and resource availability among leaves, leading to a graded but geometric size sequence [37]. Under these conditions, where extreme sun–shade dimorphism is absent, the leaf size sequence may remain relatively finely graded, potentially contributing to smaller common ratios in culms with larger numbers of leaves. Under this architectural arrangement, increasing leaf number may be accommodated by a finer gradation in leaf size rather than by a pronounced increase in size heterogeneity.

This interpretation is consistent with the findings of Meng et al. [12], who reported that for *Sasaella kogasensis* ‘Aureostriatus’, a species with leaves sparsely aggregated at the distal internodes, the geometric series provided a good fit for leaf area sequences, with MAPE values below 5% for 95.1% of the valid samples. In that study, the mean common ratio was negatively correlated with the number of leaves per culm, echoing the pattern observed in the present study. In contrast, species with vertically distributed leaves along the culm, such as maize [38] and *Semiarundinaria densiflora* [23], experience a pronounced vertical light gradient, with upper leaves receiving full sunlight and lower leaves being increasingly shaded. Such gradients may promote the development of sun leaves (smaller, thicker) at the top and shade leaves (larger, thinner) at the bottom, generating substantial variation in leaf size along the culm [39]. Such variation could obscure any intrinsic proportional relationship among adjacent leaves when ordered by size, thereby weakening the geometric series pattern and potentially weakening the association between the common ratio and the number of leaves per culm. Similarly, Shi et al. [20] reported that for *Shibataea chinensis*, a dwarf bamboo with vertically distributed leaves, the fit of the geometric series to leaf area sequences was considerably less accurate than for floral parts, with mean absolute percentage errors often approaching or exceeding 10%. The contrast between apical clustering and vertical distribution suggests a potential role of light uniformity in influencing leaf size sequences. When leaves develop under relatively uniform light conditions, as may occur in apically clustered bamboos, the geometric series model describes the size distribution more effectively, and the common ratio tends to decrease as more leaves are added to the sequence, reflecting a more finely graded size continuum. The extreme case of this pattern is observed in floral organs, where petals (or tepals) develop within a compact whorl under highly uniform environmental conditions. Deng et al. [21] found that tepal length sequences in *Magnolia × soulangeana* conformed to a geometric series with MAPE values below 1.2% for all 359 flowers examined, and Shi et al. [20] reported that over 97.8% of individual flowers from four Magnoliaceae and two Rosaceae species had MAPE values below 5% for floral part area sequences, with common ratios often much closer to unity than those observed for leaves. The near-perfect light uniformity during floral organ development likely accounts for the notably small common ratios and the remarkable precision of the geometric series fit. Thus, the negative association between the estimated common ratio and leaf number in *Indocalamus* bamboos may be associated with their apically clustered leaf architecture, which could reduce light heterogeneity and help maintain a finely graded leaf size sequence as leaf number increases.

### 4.3. Limitations and Future Directions

Despite the robust support for the geometric series hypothesis across the six *Indocalamus* species, several limitations of the present study deserve attention. First, although we have argued that the apical clustering of leaves creates a relatively homogeneous light environment, we did not quantify the above-ground architectural structure of individual culms. The branching patterns of *Indocalamus* bamboos, such as branch number, branch length, branch angle, and the vertical distribution of branches along the culm, are known to vary both within and among species. These architectural traits directly influence the spatial arrangement of leaves and, consequently, the light environment each leaf experiences. Küppers [13] emphasized that understanding the ecological significance of above-ground architectural patterns requires an integrated assessment of cost–benefit relationships, yet quantitative descriptors of such patterns remain challenging to measure. The present study did not overall quantify these architectural parameters, which may have obscured the mechanistic link between culm architecture and the observed patterns of common ratio variation. In addition, the negative relationship between the estimated common ratio and leaf number should be interpreted with caution. Because for a finite geometric sequence spanning a given size range, an increase in the number of elements can itself cause the common ratio to approach unity. In naturally varying culms, however, leaf number and the realized range of leaf lengths vary simultaneously, making it difficult to separate the mathematical effect of *n* from variation in leaf size range using the present observational data. Consequently, the negative relationship between $\hat{q}$ and leaf number should not be regarded as independent evidence for an architectural mechanism.

Second, although the six species were sampled from three geographically distinct locations (Jiangsu and Sichuan provinces, both within the subtropical zone of China), the relatively modest number of species (six out of the 22 species and six varieties recognized in *the Flora of China*, see https://www.iplant.cn/z/frps/66502; accessed on 1 August 2026) limits the generality of our conclusions. Nevertheless, the wide range of leaf number per culm (3 to 65 leaves) and the substantial variation in leaf size across species suggest that the sampled species encompass a considerable portion of the morphological variation within the genus. At the intraspecific level, the absence of replicated populations or identified genotypes prevented an assessment of within-species variation in the observed patterns. In addition, the four species sampled in Nanjing in July 2014 and the two species sampled in Sichuan in November 2024 differed in sampling location, year, season, and population, which may also have contributed to the observed differences among the sampled species groups. Furthermore, individual culms were used as the sampling and analytical units, whereas clump identity was not recorded during sampling; therefore, potential statistical dependence among culms originating from the same clump could not be explicitly evaluated. Further sampling across populations and genotypes would therefore be useful for assessing the consistency of these patterns within *Indocalamus*.

Third, our inference that apical clustering minimizes self-shading and promotes uniform leaf development is indirect; we did not directly measure light profiles within culm canopies or quantify the degree of self-shading among leaves. Future studies should incorporate high-resolution three-dimensional digitizing or LiDAR-based approaches to quantify above-ground architectural traits, such as branch angle, branch length, and the ratio of crown height to culm height. Mathematical approaches, such as the bell-shaped function used to describe the vertical distribution of leaf size metrics in maize [38], may provide a useful tool for quantifying architectural patterns in bamboos and other plant groups. Such measurements would allow the relationship among leaf number, leaf size range, culm architecture, and the estimated common ratio to be evaluated more explicitly, helping to distinguish mathematical constraints from biologically mediated variation. Additionally, extending this approach to other plant groups with similar leaf deployment patterns, particularly shrubs and herbaceous species with moderate numbers of leaves per shoot, would help assess the broader applicability of the geometric series model for describing leaf size sequences across different plant groups and architectural types. Comparisons with other bamboo taxa and plants differing in leaf deployment architecture would also help determine how strongly the observed pattern depends on the relatively clustered leaf arrangement characteristic of *Indocalamus*.

Finally, although the consistently low prediction errors support the applicability of the GS model, the present study did not compare it with alternative monotonic rank-size models or evaluate its fitting performance against simulation-based null expectations. Future studies could combine comparisons with plausible alternative rank-size models and simulation-based null models to assess whether similarly low fitting errors commonly arise from ranked samples generated by continuous distributions, thereby helping to determine the specificity of the geometric pattern detected by the GS model.

## 5. Conclusions

In the present study, we tested the geometric series hypothesis for the sorted leaf length sequence per culm in ascending order of six *Indocalamus* bamboo species. A total of 577 culms were retained for final analysis after excluding culms with fewer than three or more than 90 leaves, and those with estimated common ratios exceeding 1.2. The geometric series model provided an excellent fit for the majority of culms, with MAPE values below 5% for 75.4% of the culms and below 10% for 96.5% of the culms. The estimated common ratios ranged from 1.01 to 1.19, with a mean of 1.05, indicating a modest but consistent geometric increase in leaf length along the ascending size sequence, with each successive leaf longer than the previous by a roughly constant factor. The estimated common ratio and minimum leaf length also differed among the six sampled species groups. Significant negative associations between the estimated common ratio and leaf number were consistently detected within all six species, with culms bearing more leaves generally having smaller common ratios. These findings provide strong empirical support for the geometric series as a robust descriptor of leaf length organization in the sampled *Indocalamus* bamboos. Extension of this approach to other plant groups with similar leaf deployment patterns, particularly those with moderate numbers of leaves per shoot, would help assess the broader applicability of the geometric series model for describing ordered leaf size sequences under relatively uniform light environments.

## Figures and Tables

**Table 1:** Model-fitting performance for the original and sensitivity-analysis datasets.

Group	Sample Size	Mean $\hat{q}$	Range of $\hat{q}$	Mean MAPE (%)	MAPE < 5% (%)	MAPE < 10% (%)
Primary analysis dataset	577	1.0534	1.0110–1.1912	3.58	75.39	96.53
Including $\hat{q}$ > 1.2	592	1.0599	1.0110–1.7238	3.65	74.66	95.95
Including *n* > 90	578	1.0533	1.0110–1.1912	3.59	75.26	96.54
Including both exclusions	593	1.0598	1.0110–1.7238	3.65	74.54	95.95

## Data Availability

The data can be found in Supplementary Table S1 online.

## Supplementary Materials

The following supporting information can be downloaded at https://www.mdpi.com/article/10.3390/plants15203129/s1: Figure S1: Distributions of leaf number per culm and estimated common ratio used for sample screening; Table S1: Leaf size data of six *Indocalamus* species.

## Appendix A

Here we show an example of fitting a leaf length sequence using the “fitGS” function in the “biogeom” package (version 1.5.2) in R (version 4.3.3):

install.packages(“biogeom”)

library(biogeom)

L <- c(11.6, 21, 15.2, 15.5, 22.8, 26.1, 20.4, 17.9, 16.2)

Res <- fitGS(L)

names(Res)

Here, L is the numeric vector saving the leaf (lamina) length data in cm of an *Indocalamus* culm.

## References

[1] Niklas K.J. (1994). Plant Allometry: The Scaling of Form and Process.

[2] Falster D.S., Westoby M. (2003). Leaf size and angle vary widely across species: What consequences for light interception?. New Phytol..

[3] Shi P., Li Y., Niinemets Ü., Olson E., Schrader J. (2021). Influence of leaf shape on the scaling of leaf surface area and length in bamboo plants. Trees.

[4] Baird A.S., Taylor S.H., Pasquet-Kok J., Vuong C., Zhang Y., Watcharamongkol T., Scoffoni C., Edwards E.J., Christin P.-A., Osborne C.P. (2021). Developmental and biophysical determinants of grass leaf size worldwide. Nature.

[5] Milla R., Reich P.B. (2007). The scaling of leaf area and mass: The cost of light interception increases with leaf size. Proc. R. Soc. B Biol. Sci..

[6] Niklas K.J., Cobb E.D., Niinemets Ü., Reich P.B., Sellin A., Shipley B., Wright I.J. (2007). “Diminishing returns” in the scaling of functional leaf traits across and within species groups. Proc. Natl. Acad. Sci. USA.

[7] Shi P., Liu M., Ratkowsky D.A., Gielis J., Su J., Yu X., Wang P., Zhang L., Lin Z., Schrader J. (2019). Leaf area–length allometry and its implications in leaf shape evolution. Trees.

[8] Yu X., Shi P., Schrader J., Niklas K.J. (2020). Nondestructive estimation of leaf area for 15 species of vines with different leaf shapes. Am. J. Bot..

[9] Schrader J., Shi P., Royer D.L., Peppe D.J., Gallagher R.V., Li Y., Wang R., Wright I.J. (2021). Leaf size estimation based on leaf length, width and shape. Ann. Bot..

[10] Sun J., Wang M., Lyu M., Niklas K.J., Zhong Q., Li M., Cheng D. (2019). Stem and leaf growth rates define the leaf size vs. number trade-off. AoB Plants.

[11] Koyama K., Smith D.D. (2022). Scaling the leaf length-times-width equation to predict total leaf area of shoots. Ann. Bot..

[12] Meng Y., Ratkowsky D.A., Yao W., Heng Y., Shi P. (2025). The geometric series hypothesis of leaf area distribution and its link to the calculation of the total leaf area per shoot of *Sasaella kongosanensis* ‘Aureostriatus’. Plants.

[13] Küppers M. (1989). Ecological significance of above-ground architectural patterns in woody plants: A question of cost-benefit relationships. Trends Ecol. Evol..

[14] Bendel R.B., Higgins S.S., Teberg J.E., Pyke D.A. (1989). Comparison of skewness coefficient, coefficient of variation, and Gini coefficient as inequality measures within populations. Oecologia.

[15] Huang L., Ratkowsky D.A., Hui C., Gielis J., Lian M., Yao W., Li Q., Zhang L., Shi P. (2023). Inequality measure of leaf area distribution for a drought-tolerant landscape plant. Plants.

[16] Weiner J., Solbrig O.T. (1984). The meaning and measurement of size hierarchies in plant populations. Oecologia.

[17] Lian M., Shi P., Zhang L., Yao W., Gielis J., Niklas K.J. (2023). A generalized performance equation and its application in measuring the Gini index of leaf size inequality. Trees.

[18] Lian M., Chen L., Hui C., Zhu F., Shi P. (2024). On the relationship between the Gini coefficient and skewness. Ecol. Evol..

[19] Jiao Z., Liu S., Niklas K.J., Damgaard C.F., Yao W., Lian M., Jiang F., Shi P. (2025). Linking the Weibull distribution to Gini coefficients: A bamboo specific framework for intra-culm leaf area inequality. Front. Plant Sci..

[20] Shi P., Li B.L., Wang J., Mu Y., Yao W., Lian M., Deng L., Niklas K.J. (2025). Geometric series exists in nature: Evidence from sorted area sequences of floral parts and leaves. Ann. N. Y. Acad. Sci..

[21] Deng L., Wang J., Zhang L., Hölscher D., Shi P. (2025). Testing the validity of the Montgomery–Koyama–Smith equation and the power law equation using 3231 tepals of a *Magnolia* species. Trees.

[22] Shi P., Xu Q., Sandhu H.S., Gielis J., Ding Y., Li H., Dong X. (2015). Comparison of dwarf bamboos (*Indocalamus* sp.) leaf parameters to determine relationship between spatial density of plants and total leaf area per plant. Ecol. Evol..

[23] Li Z., Niklas K.J., Wang J., Deng L., Huang W., Shi P. (2026). Scaling relationship between total leaf size and total number of leaves per culm of *Semiarundinaria densiflora*. Ann. Bot..

[24] Yan C., Shi P., Yao W., Yu K., Niinemets Ü. (2025). A nonlinear fitting method provides strong support for geometric series of stomatal area in 12 Magnoliaceae species. Plants.

[25] Nelder J.A., Mead R. (1965). A simplex method for function minimization. Comput. J..

[26] Shi P., Gielis J., Quinn B.K., Niklas K.J., Ratkowsky D.A., Schrader J., Ruan H., Wang L., Niinemets Ü. (2022). ‘biogeom’: An R package for simulating and fitting natural shapes. Ann. N. Y. Acad. Sci..

[27] R Core Team (2023). R: A Language and Environment for Statistical Computing.

[28] Hsu J. (1996). Multiple Comparisons: Theory and Methods.

[29] Lin S., Zhang L., Reddy G.V.P., Hui C., Gielis J., Ding Y., Shi P. (2016). A geometrical model for testing bilateral symmetry of bamboo leaf with a simplified Gielis equation. Ecol. Evol..

[30] Xu J., Niklas K.J., Zhou D., Gu W., Shi P. (2026). Validation of the geometric series hypothesis using individual and pooled samples of *Malus* cultivar flowers. Ann. Bot..

[31] Su J., Niklas K.J., Huang W., Yu X., Yang Y., Shi P. (2019). Lamina shape does not correlate with lamina surface area: An analysis based on the simplified Gielis equation. Glob. Ecol. Conserv..

[32] Tsaniklidis G., Makraki T., Papadimitriou D., Nikoloudakis N., Taheri-Garavand A., Fanourakis D. (2025). Non-destructive estimation of area and greenness in leaf and seedling scales: A case study in cucumber. Agronomy.

[33] Kim G.-T., Tsukaya H., Saito Y., Uchimiya H. (1999). Changes in the shapes of leaves and flowers upon overexpression of cytochrome P450 in *Arabidopsis*. Proc. Natl. Acad. Sci. USA.

[34] Kim G.-T., Shoda K., Tsuge T., Cho K.-H., Uchimiya H., Yokoyama R., Nishitani K., Tsukaya H. (2002). The *ANGUSTIFOLIA* gene of *Arabidopsis*, a plant *CtBP* gene, regulates leaf-cell expansion, the arrangement of cortical microtubules in leaf cells and expression of a gene involved in cell-wall formation. EMBO J..

[35] Satterlee J.W., Scanlon M.J. (2019). Coordination of leaf development across developmental axes. Plants.

[36] Kim G.-T., Tsukaya H., Uchimiya H. (1998). The *ROTUNDIFOLIA3* gene of *Arabidopsis thaliana* encodes a new member of the cytochrome P-450 family that is required for the regulated polar elongation of leaf cells. Genes Dev..

[37] Fu Q., Shi P., Gielis J., Niklas K.J. (2025). Non-destructive prediction of shoot-level leaf area and biomass in *Indocalamus* bamboo via scaling laws. Front. Plant Sci..

[38] Fan P., Li Y., Evers J.B., Ming B., Wang C., Li S., Xie R. (2020). A new empirical equation to describe the vertical leaf distribution profile of maize. J. Agric. Sci..

[39] Clark L.G., Londoño X., Ruiz-Sanchez E., Liese W., Köhl M. (2015). Bamboo taxonomy and habitat. Bamboo: The Plant and Its Uses.

